# Influence of Compost Amendments on the Composition of Pistachio Nuts in Young Pistachio Trees (*Pistacia vera* L.)

**DOI:** 10.3390/foods15040697

**Published:** 2026-02-13

**Authors:** Marta I. Saludes-Zanfaño, M. Remedios Morales-Corts, Louise Ferguson, Ana M. Vivar-Quintana

**Affiliations:** 1 Plant Production Group, Faculty of Environmental and Agricultural Sciences, Universidad de Salamanca, 37007 Salamanca, Spain; reme@usal.es; 2 Department of Plant Sciences, University of California Davis, One Shields, Avenue, Davis, CA 95616, USA; lferguson@ucdavis.edu; 3 Food Technology Group, Superior Polytechnic School of Zamora, Universidad de Salamanca, 49022 Zamora, Spain; avivar@usal.es

**Keywords:** compost, compost tea, organic fertilization, nut quality, sustainable agriculture

## Abstract

New fertilization strategies in pistachio cultivation are necessary to reduce the environmental impact of mineral fertilization. The influence of organic fertilization strategies, compost and compost tea, on the growth, yield, and chemical composition of pistachio nuts (*Pistacia vera* L.) was investigated for two sequential seasons in Spain. Conventional synthetic fertilizers were compared with three organic treatments: compost (T1), compost with compost tea (T2), and compost tea combined with mineral fertilizers (T3). Organic fertilization, alone or combined with mineral fertilization, proved adequate for tree growth and production. The nut composition including mineral content, fatty acid profile, and amino acid composition, was comparable to that obtained with mineral fertilization. Linolenic acid content was higher in treatments that incorporated compost tea (T2 and T3). No changes were observed in mineral composition except for the Zn, which was higher in T3 during one season. Differences in pistachio nut composition between growing seasons were more pronounced than those associated with the fertilization treatments. This is probably a result of alternate bearing. In this trial, integrated organic–mineral fertilization of young pistachio trees reduced the need for mineral fertilization.

## 1. Introduction

Pistachio is a profitable tree crop. Since 2000 new acreage has increased sharply in California, Spain and other countries [1]. California now has more than 197,000 hectares (488,000 acres) [2], and Spain than 70,000 hectares [3] The combination of increased ingredient use and recently demonstrated health benefits are among the drivers of this industry expansion [4].

Pistachios have high levels of monounsaturated and polyunsaturated fatty acids [5]. They are approximately 20% of vegetable protein [6]. Their total amino acid ratio is higher than most nuts including almond, walnut, pecan and hazelnut [7]. They are low in carbohydrates, high in fiber [8], and contain the essential micronutrients calcium, potassium, phosphorus, iron and copper [9]. They contain high levels of phytosterols, carotenoid phenolic acids and gamma tocopherols [10]. Cultivar origin, ecological conditions, and cultivation practices have been reported to significantly affect the kernel protein, fat, dry matter and ash content [11,12]. Meimand et al. [13] reported yield and particularly quality, are partially a function of nutrient management.

Pistachio requires adequate fertilization for tree growth and economically viable yields. However, the production and use of synthetic fertilizers contribute significantly to greenhouse gas (GHG) emissions. The energy to manufacture fertilizers and the release of nitrous oxide (N_2_O) following soil application [14] are the primary sources of GHG in pistachio production. Potentially, high-quality organic alternatives, such as Class A composts, could be used to supplement conventional synthetic fertilizers, reducing the crop production footprint [15]. Composting biologically transforms waste, allowing it to stabilize and be used as fertilizer [16].

Life cycle assessment studies conducted in pistachio orchards have demonstrated that conventional production systems relying on mineral fertilizers are associated with substantial emissions of CO_2_, N_2_O and CH_4_, identifying chemical inputs—particularly fertilizers—together with fuel and electricity, as major contributors to total GHG emissions in pistachio production systems [17]. Additional benefits include an increase in soil organic matter, together with improvements in soil structure and water-holding capacity [18,19,20,21]. Aslan et al. [22] reported a combination of mineral and organic fertilization increased yield by 40%, suggesting that the organic amendments increased the efficiency of mineral fertilizers. Similarly, Paymaneh et al. [23] demonstrated vermicompost application promoted increased pistachio growth and nutrient uptake under adequate irrigation conditions.

Despite these encouraging findings, knowledge remains limited regarding the effects of organic fertilization on nut composition, particularly the use of compost tea. Compost tea is defined as a liquid extract obtained from mature compost by suspension in water. It is applied as a soil drench or foliar spray, providing soluble nutrients and organic compounds in a readily available form [15,18]. However, its influence on nut quality, including fatty acid and amino acid profiles, has not been sufficiently elucidated.

After bloom and fruit set the early vegetative stages, pistachio trees prioritize canopy expansion, leaf area development, and the establishment of an efficient photosynthetic system, processes closely linked to nutrient availability and uptake. At this stage, nutrient demand—particularly nitrogen—is primarily associated with active vegetative growth and depends largely on current-season uptake rather than on stored reserves, making young trees especially sensitive to nutrient availability and timing [24]. Organic amendments such as compost may contribute to a gradual release of nutrients while improving soil organic matter content, soil structure, and microbial activity, which are key factors influencing nutrient retention and availability in orchard soils. On the other hand, compost tea provides a more readily available nutrient fraction that may temporarily enhance nutrient uptake during periods of rapid vegetative growth. Despite their increasing use in sustainable orchard management, information on the individual and combined effects of these amendments on early vegetative development of young pistachio trees under field conditions remains limited.

This study examined the effects of compost and compost tea, applied alone or in combination with mineral fertilizers, on early vegetative growth, yield components, and kernel nutritional quality of young pistachio trees under field conditions. The main objective was to determine whether these organic conditioners can serve as sustainable alternatives to conventional fertilization without compromising tree yield or nut quality.

## 2. Materials and Methods

### 2.1. Experimental Design

The experimental site was located in Parada de Rubiales, Salamanca (Spain), at 41°9′18.02″ N, 5°26′50.24″ W, and 844 m above sea level. The study was conducted over two production seasons with 400 mm annual rainfall and 12.6 °C annual medium temperature and 471 mm and 12.0 °C in 2022 and 2023 respectively. Soils were a calcareous Cambisol, with loam–clay–sand texture and a pH value of 8.1. The orchard was planted in 2018 with a ‘Kerman’ (*Pistacia vera* L.) female scion budded onto a ‘UCB1’ hybrid (*P. atlantica* Desf. × *P. integerrima* J.L. Stewart) rootstock. The spacing was 4.60 × 4.60 m, 374 trees per hectare. Male trees were planted every third row and eights tree, 11% males and 337 producing trees per hectare. A drip system with 2 drippers per tree delivered 4 L per hour.

The experimental design was four randomized complete blocks. Four fertilization treatments were evaluated. Within each experimental block, treatments were represented by either four or five trees, depending on tree availability and uniformity in the orchard. The distribution of trees within blocks was adjusted to maintain the same total number of experimental units per treatment. Consequently, each treatment included 18 trees overall, resulting in a total of 72 trees in the entire experiment. Each parameter was determined in all the trees during the two consecutive campaigns. The 4 treatments were a synthetic fertilizer control, 240 g tree^−1^ 20-6-6 (NPK) (Commercial name: DECODER TOP 1) in the spring. Treatment 1 (T1) was 5 kg tree^−1^ of compost in the beginning of fall (2-0.2-2 NPK considering half mineralization in the campaign). Treatment 2 (T2) consisted of 5 kg tree^−1^ of compost applied in early autumn, combined with compost tea applications. Compost tea was applied to the soil in two equal doses of 333 mL tree^−1^ each, the first at early vegetative development (bud break) and the second four weeks later during the period of active shoot growth. In addition, a single foliar application of compost tea (333 mL tree^−1^) was performed at the onset of fruit filling. Treatment 3 (T3) consisted of compost tea applied to the soil and foliage following the same application scheme described for T2, combined with mineral fertilization (240 g tree^−1^ of 20–6–6 NPK) applied in spring. The compost tea was diluted 1:3 (*v*/*v*, compost tea: water) for soil applications and 1:5 (*v*/*v*) for foliar application. In treatments T2 and T3, compost tea was applied to the soil in two split applications. The first soil application was carried out during early vegetative development (bud break), while the second was performed four weeks later, coinciding with the period of active shoot growth. In addition, a single foliar application of compost tea was applied at the beginning of fruit filling. The total nutrient contents received by tree and treatment were Control (48 g N, 12 g P, 12 g K), T1 (50 g N, 5 g P, 50 g K), T2 (53 g N, 5,1 g P, 53,8 g K) and T3 (51 g N, 12,1 g P, 15,8 g K). Weather conditions (Temperature, Humidity, Wind speed, Radiation and Precipitation) during 2022 and 2023 were recorded daily from a weather station located near the experimental orchard. (Appendix A, Table A1).

### 2.2. Compost and Compost Tea Production and Composition

The compost was produced from pruning residues and green waste collected from public gardens in the province of Salamanca (Spain). Composting was carried out at a nursery facility located in Salamanca (40°57′23″ N, 5°41′8″ E). The waste was shredded and arranged in piles measuring 15 m in length, 2 m in width, and 2 m in height. During composting, the piles were turned twice a week for the first eight weeks and subsequently once a week. Moisture and temperature were recorded weekly, and the process was completed after 180 days. The main physicochemical properties of the compost were: Organic Matter: 41%, E.C.: 1.74, pH: 7.6, N total: 2.1%, P_2_O_5_: 1901 mg kg^−1^, K_2_O: 14,993 mg kg^−1^ and CaO: 20,119 mg kg^−1^. To produce compost tea (CT) from gardening waste, a 1:5 (*v*/*v*) ratio was used with tap water, aerated for 5 h per day with a 750 W pump at 300 rpm. It was filtered after one week. Subsequently, a physicochemical analysis of the extract was conducted. Table 1 presents the physicochemical properties of the compost tea (CT) employed in the trial. The CT exhibited a slightly basic pH (7.32) and high levels of NO^3−^ and K_2_O. Additionally, it contained high concentrations of humic acids. The compost tea was prepared following the methodology described by Morales-Corts et al. [25] and their composition was analyzed after preparation.

Compost and compost tea compositions are reported separately because compost tea is a liquid extract that reflects only the water-extractable fraction of the solid compost and does not represent its bulk composition; therefore, their physicochemical properties are not directly comparable [18,25]. In this study, compost and compost tea were considered distinct organic amendments due to their different physicochemical characteristics, modes of application, and expected mechanisms of action. Accordingly, they were evaluated both individually and in combination within the experimental design.

### 2.3. Agronomic Surveys

Throughout the experimental period, tree growth and development were monitored, including chlorophyll content (SPAD-502 chlorophyll meter, Konica Minolta, Tokyo, Japan), annual shoot length (cm), and trunk diameter growth (cm). Trunk girth was measured annually in October at a fixed height of the midpoint be-tween the junction of the primary scaffold branches (branch crotch) and the soil surface. Measurements were taken on the same 18 trees per treatment to ensure consistency across seasons and treatments on overall tree growth. The SPAD-502 measurements were conducted in the field between 10:00 and 12:00 h. During measurements, the adaxial side of the leaves was consistently placed toward the instrument’s emitting window, ensuring that major veins were avoided obtaining accurate readings. A total of 36 fully mature leaves were selected for analysis. At the start of the flowering season, 16 shoots were randomly selected and tagged on each tree. These tagged shoots were measured for annual shoot length at both the beginning of the flowering season and at the end of the growing season in 2022 and 2023. Trunk girth was measured annually in October at the midpoint between the tree’s crotch and the ground, (give and approximate height from the ground) on the same 18 trees per treatment, to assess the effect of treatments on overall tree growth.

In addition, all fruits from each tree were harvested individually to evaluate the effect of the fertilization treatments on yield. The fresh weight per tree was subsequently determined using a certified commercial scale.

### 2.4. Compositional Analysis of Pistachio Nuts

For compositional analyses, three independent composite kernel samples were prepared per treatment and growing season. Each composite sample was obtained by pooling pistachio nuts collected from the same 18 trees per treatment. Pistachio nuts were harvested at commercial maturity each growing season (2022 and 2023). All samples were processed and analyzed within three weeks after harvest. After shelling, kernels were dried to 6% moisture, ground, and homogenized. From each composite sample, a 150 g subsample was taken for the compositional analyses. Following grinding, samples were maintained under controlled, light-protected conditions until analysis. Samples from each growing season were analyzed in their corresponding year.

#### 2.4.1. Proximate Composition

The content of carbohydrates, moisture, protein, fat, starch, sugars, fibber, and ash was analyzed. Crude protein content (N × 6.25) was measured using the macro-Kjeldahl procedure. Crude fat was measured by Soxhlet extraction with petroleum ether using a known sample mass. Moisture content was determined by the AACC 14-15 A method and ash content was measured at 550 ± 10 °C using the AACC method 08-01.01. Total dietary fiber was analyzed using AOAC method 991.43 with an ANKOM dietary fiber analyzer (ANKOM technology, New York, NY, USA). Total carbohydrate content was calculated by difference ((100 − (Ash + Protein + Total Fat)), and the energy content (kcal/100 g) was estimated using conversion factors of 4 kcal/g for protein and carbohydrates and 9 kcal/g for fat.

#### 2.4.2. Mineral Composition

Nutrient concentrations (Mg, Ca, Fe, Zn) were determined by ICP-MS. Prior to the analysis of the mineral elements, the samples (50 mg of ground pistachio) were mineralized in a microwave system (Milestone ULTRAWAVE, Sorisole (BG), Italy) with 5 mL of HNO_3_ and a power of 1000 W was applied for 5 min. Then, an additional 5 mL of HNO_3_ and 1 mL of H_2_O_2_ (30%) were added, applying a power of 1000 W for 10 min. The sample was cooled to room temperature and prepared to 100 mL with distilled water. Elemental concentrations were measured using an Agilent 7800 inductively coupled plasma mass spectrometer (Agilent, Santa Clara, CA, USA). Instrumental operating conditions included an RF power of 1550 W, a plasma air flow rate of 15 L/min, an auxiliary air flow 0.9 L/min, and a Nebulizer air flow 1.12 L/min. Quantification was carried out using certified standard solutions (1 g/L) supplied by Panreac (Castellar del Vallès, Spain). Calibration curves applied are shown in Appendix A (Table A2).

#### 2.4.3. Amino Acid Composition

Amino acid determination was performed following the method described by Garcia-Palmer et al. [26] with minor modifications, using 0.2 g of sample homogenized in 6 N hydrochloric acid (HCl). Then, sample is heated in an oven 110 °C for 23 h. The hydrolysate is diluted properly and filtered by 0.45 µm filter prior chromatography analysis.

For amino acid derivatization, the hydrolyzed samples were automatically derivatized with o-ftalaldehído (OPA) (Agilent Tecnologies Application note 5980-1193). After derivatization, each sample was injected onto a Zorbax Eclipse-AAA column, 3.5 μm, 150 × 4.6 mm (Agilent), with detection at λ = 338 nm. Mobile phase A was NaH_2_PO_4_ 40 mM, adjusted to pH 7.8 with NaOH, while mobile phase B was acetonitrile/methanol/water (45/45/10 *v*/*v*/*v*/*v*). Separation was achieved at a flow rate of 2 mL/min, using a gradient of 0–1 min 0% B, 9.8 min 57% B, 10 min 100% B, and 12 min 100%. An Agilent Infinity 1260 HPLC (Agilent Technologies, Santa Clara, CA, USA) coupled to a diode array detector (UV wavelength set at 338 nm) was used. Quantification was performed using an amino acid standard according to the methodology defined in Commission Regulation (EC) No 152/2009 (European Commission: Brussels, Belgium, 2009). The peak areas of both the sample and the standard are measured for each amino acid, and the corresponding amount is calculated in grams of amino acid per kilogram of sample.

Where:

$$x=\frac{A\times C\times M\times V}{B\times m\times 1000}$$

A = Peak area of the hydrolysate or extract

B = Peak area of the calibration standard solution

M = Molecular weight of the amino acid being determined

C = Concentration of the standard in μmol/mL

m = Weight of the sample (g)

V = Total calculated dilution volume of the extract (mL)

#### 2.4.4. Fatty Acid Composition

Fatty acid analysis was performed by gas chromatography (GC) after sample extraction as described in the method of Bligh and Dyer [27]. For this purpose, 1 g of sample was mixed with 4.5 mL of ultrapure H_2_O and 22.5 mL of chloroform:methanol (1:2, *v*/*v*). After stirring for 2 min, 7.5 mL of water and 7.5 mL of CHCl_3_ were added and stirred again. Afterwards, it was centrifuged for 5 min at 5000 r.p.m. and the upper phase was extracted and dried in a rotary evaporator at 30 °C. From the extracted fat, 0.2 g was used for methylation. Four milliliters of hexane and 0.3 mL of KOH (2M) were added and stirred. After resting at room temperature for 30 min, 1.5 mL of the upper phase was collected for fatty acid methyl ester analysis. A Bruker GC-FID (SCION 456-GC, SCION Instruments, Didcot, UK) and a DB-225ms column (30 m, 0.250, 0.25 µm) from Agilent Technologies were used, with N2 as the carrier gas. The temperature program was 80 °C for 2 min, followed by an increase of 5 °C/min to 180 °C, which was maintained for 5 min. Subsequently, at a rate of 1 °C/min, the sample was initially heated to 200 °C and subsequently brought to 240 °C at 10 °C/min, which was maintained for 3 min. Fatty acids were identified by comparing retention times with a commercial Supelco FAME standard mixture of 37 components. The results were expressed as percentage of total fatty acid methyl esters.

### 2.5. Statistical Analysis

Assumptions of normality and homoscedasticity were assessed prior to analysis of variance (ANOVA) to validate the statistical approach. Normality was evaluated using the Shapiro–Wilk test, whereas homogeneity of variances was assessed by Levene’s test. When these assumptions were not met, data transformations were considered; however, no significant deviations were observed, allowing the application of ANOVA followed by Tukey’s HSD test for multiple comparisons. All statistical analyses were performed using SPSS version 27.0, with a significance level set at *p* < 0.05. An unsupervised pattern recognition analysis using principal component analysis (PCA) was applied. The number of PCs for classification purposes was determined by selecting those variables with an eigenvalue > 1. The data were analyzed using IBM SPSS Statistics (version 27.0).

## 3. Results

### 3.1. Growth-Production Parameters and Organic Fertilization Incidence

Tree growth-production parameters (Table 2) were evaluated during the two seasons (2022–2023) in young pistachio trees. No statistically significant differences were observed in trunk diameter growth across treatments in either growing season. In contrast, shoot length and yield-related parameters exhibited statistically significant differences only in one of the two evaluated seasons, indicating that treatment effects on these variables were season-dependent and strongly influenced by interannual variability. Similarly, shoot length and yield per tree showed differences, but only in one of the seasons studied.

The clear differences observed between the two growing seasons indicated that year-to-year environmental variability exerted a stronger influence on tree performance than the fertilization treatments themselves. Although 2022 presented the lowest yields across treatments (Table 2), the meteorological records showed that it was 2023 that had less favorable climatic conditions for pistachio development, with lower spring–summer rainfall and higher temperatures (Table A1). The cumulative results suggested that all the treatments replacing or supplementing mineral fertilization with organic fertilization supplied sufficient nutrition for young pistachio trees.

The comparatively milder pattern in 2022 may have favoured more regular early vegetative activity, while the drier and warmer conditions in 2023 appeared to have limited vegetative growth and altered the allocation of assimilates, contributing to the different seasonal patterns recorded in terms of shoot length and nut weight. Taken together, these findings reinforce the idea that the climatic context of each season outweighed the effect of the fertilisation strategy. Shoot expansion and nut development in young pistachio trees depend largely on adequate soil moisture and moderate temperatures during the early stages of growth, so that environmental fluctuations can dominate tree responses during the first years of production [24]. In this study, the contrast between a cooler year with more uniform irrigation (2022) and a drier, warmer year (2023) offers a plausible explanation for the seasonal variation observed in growth and yield parameters.

### 3.2. Proximate Composition

The proximate composition (g 100 g^−1^ dry weight, dw) of pistachios in each harvest season are shown in Table 3. Fats, ranging from 48.06 to 52.26 g 100 g^−1^, were the major pistachio constituents detected. Protein and carbohydrates followed at 22.28 and 25.18 g 100 g^−1^ and 21.58 and 24.13 g 100 g^−1^ respectively.

The results obtained for the kernels from the young trees analyzed are similar to those described in the literature for fat [7,28] and protein [12]. However, our pistachios had a higher ash content, carbohydrates and energy [29,30] and a lower sugar content [31].

The proximate composition of the pistachio kernels varied across both treatments and growing seasons (Table 3). It can be observed a strong influence of the growing season on fat, protein, starch, sugars, ash and energy content. This marked seasonal effect indicates that the climatic conditions prevailing during each cycle, especially temperature distribution and rainfall, played a decisive role in nutrient allocation to the kernels. Similar annual fluctuations in pistachio composition have been described previously by Rabadán et al. [32] and Gündeşli [31] indicating variations with crop year for protein, fiber, carbohydrate, ash and energy parameters.

In 2022, a season characterized by lower yield per tree (Table 2), kernels showed higher concentrations of protein, sugars and ash, together with lower fat and starch contents. This inverse seasonal pattern suggests differences in assimilate partitioning between years and is therefore interpreted as reflecting interannual variability. Similar seasonal trends in kernel composition across contrasting production years have been reported previously in pistachio by Okay [33] and Gündeşli [31]. The contrasting climatic scenario in 2023, with reduced summer rainfall and higher temperatures (Table A1), likely altered carbohydrate partitioning and nutrient assimilation, contributing to the interannual variability found in the present study.

Compost (T1) consistently produced higher fat content in both seasons. This result is plausible considering that compost can improve soil structure and nutrient retention, in turn promoting root physiological activity and the synthesis of lipids, the principal reserve component of pistachio kernels. This mechanism is consistent with the benefits of compost-amended soils described by Benito et al. [19] and Moretti et al. [20]. Energy content followed the same pattern, as expected, given the high contribution of fat to the caloric value of nuts.

The lower sugar concentration observed in T2 during 2022 may be related to early-season effects of compost tea applications. Compost tea contains readily available nutrients and humic compounds [25], which can influence metabolic dynamics during fruit development. However, this effect was not observed in 2023, reinforcing the overriding contribution of climate on carbohydrate metabolism, consistent with Rabadán et al.’s observations [32].

For ash content, T3 (compost tea combined with mineral fertilization) had the highest value, significantly, in 2023. The elevated mineral content in this treatment is consistent with the high micronutrient levels —particularly Zn—present in the compost tea used (Table 1). These findings agree with Paymaneh et al. [23], who showed improved micronutrient uptake in pistachio under organic fertilization. The increase in ash content observed in T3 therefore likely reflects improved mineral bioavailability under mixed organic–mineral fertilization.

When considering the two seasons together, the results showed that fat and energy contents were significantly enhanced by compost fertilization (T1), while ash content was more strongly influenced by T3. Even so, the magnitude of the fertilization effects remained modest compared with the pronounced interannual differences. Environmental variability between years was more influential than the fertilization strategy Rabadán et al. (2019) [32].

Overall, the results indicate that organic fertilization, either alone or in combination with mineral fertilizers, can sustain kernel nutritional quality comparable to that obtained under conventional fertilization. Given that kernel composition largely depends on cultivar, environment and cultural practices (Tsantili et al., 2010; Seferoglu et al., 2006; Ghaseminasab et al., 2015) [11,12,30], our findings reinforce the idea that young pistachio trees respond primarily to climate while organic fertilization provides sufficient nutrient availability without compromising nut quality.

### 3.3. Mineral Composition

Table 4 shows the Mg, Ca, Fe and Zn contents of pistachio for the different treatments. The results indicate that pistachio kernels are characterized by a high Mg content (1114–1198 mg kg^−1^). Ca (1032–1216 mg kg^−1^) had the highest concentrations among the analyzed minerals in both years. These results are within the maximum and minimum values reported by Harmankaya et al. [34] with Ca values between 1614 to 3226 mg/kg and Mg between 1716 to 2402 mg/kg. Similar results have been reported by Ghaseminasab et al. [30] with Mg and Ca as the major minerals in pistachio composition. However, in Italian pistachios D’Evoli et al. [35] and in Turkish pistachios Çınar Okay [36] described P and K as the major minerals in pistachio. These differences among authors may be related to pistachio variety, production region, and whether the results are expressed in dry or fresh weight. In the present experiment, K and P were supplied through fertilization treatments and were present in the compost and compost tea applied. The mineral analysis of pistachio kernels focused on selected elements (Mg, Ca, Fe and Zn) considered more sensitive to management-induced variability.

Among the microelements, Fe contents (38.15–55.60 mg kg^−1^) were higher than Zn (12.65–23.56 mg kg^−1^), for all treatments, in both years. These values are in the ranges described by Harmankaya et al. [34] with Fe values between 38.44 and 47.26 mg/kg and Zn between 15.79 and 24.59 mg/kg. Moreover, Fe has been described as one of the most abundant minerals in Iranian pistachio [37]. Fruit mineral composition is influenced by multiple factors, such as cultivar, rootstock, fruit maturity, and harvest timing [30], so there is a wide variation in the results of mineral composition of pistachio among different researchers.

The results in Table 3 show that there are differences depending on the fertilization treatment applied each growing season. In the 2022 season, no statistically significant differences were observed among treatments for Ca, Mg and Fe content. However, the Zn concentration was higher when organic fertilization is incorporated. It was statistically significant for T3, which combines organic and mineral fertilization. On the other hand, in the 2023 season, only Ca shows differences between treatments with significantly higher concentrations when compost tea was added. Also, although there are no significant differences, the Zn content was higher in the organic fertilization treatments in 2023.

This is probably an effect of the compost tea with its particularly high Zn content (Table 1). Previous studies have shown that Zn uptake in pistachio trees is higher when compost and vermicompost are applied, due to the greater availability of this mineral in the soil [23]. The application of compost tea also resulted in a higher Ca concentration in the kernel in the 2023 season. No results have been found on pistachio kernels in this respect, however organic fertilization has been shown to increase Ca content in other nuts such as hazelnuts [38].

The Ca, Fe and Zn contents were lower in 2023. This is consistent with the lower ash value found in pistachios (Table 3). For these three minerals the growing season has been shown to be significant (*p* < 0.001). However, Mg was not influenced by the season. These results agree with Rabadán et al. [32] who reported that the contents of Ca, Zn and Fe in pistachio are mainly influenced by the environmental conditions associated with the year of cultivation. At the same time, these authors pointed out that the Mg contents show less variability due to the year. Once again, climatic factors must be accounted for to explain the mineral composition results. The results obtained indicate that mineral concentrations were higher in the year 2022, coinciding with the higher contents of protein, sugar and ash already described. As previously mentioned, the difficult environmental conditions in the year 2023 appeared to influence the mineral composition of the pistachio nuts. As Rabadán et al. [32] suggested, differences in monthly temperature and rainfall patterns can lead to significant changes in the proximate components of nuts.

### 3.4. Fatty Acid Profile

The fatty acid profile of the treated pistachios is shown in Table 5. A high concentration of unsaturated fatty acids, mainly oleic and linoleic, was found in all samples. The oleic and linoleic acids together make up over 70% of the total fatty acid content. These results agree with earlier studies by Catalán et al. [39] and Roncero et al. [40], reporting that unsaturated fatty acids constitute more than 50% of the total lipids in pistachio nuts. The unsaturated fatty acids (linoleic and oleic acids) have been described as the most abundant in pistachio [41]. The oleic acid content of our samples ranged from 40.97 to 49.42%, which is lower than that reported for Italian (70.1–71.5%), Turkish (67.62–70.41%) and Iranian (51.8–71.23%) pistachios [11,42,43]. The contents of linoleic acid (31.35–32.52%) fall within the wide range reported for Iranian pistachio varieties (17.36–35.16%) and are higher than the values reported for Turkish varieties (16.70–18.70%) [43,44]. The concentration of linolenic acid, although a minor acid, exceeded 0.6% and was higher than values described in other studies [43] which suggest higher values of this fatty acid for the Kerman variety. The large differences found between different studies have been attributed to cultivar-dependent variation [28] and to the influence of plant water status on fatty acid composition [45].

The saturated fatty acid profile was dominated by palmitic acid (C16:0), whereas oleic acid (C18:1) was the major monounsaturated fatty acid, followed by palmitoleic acid (C16:1). Similar results have been described by Okay [33] and Acar et al. [44].

The oleic/linoleic acid (O/L) ratio is commonly used as an indicator of fat quality and has been associated with oil stability in the literature. The values recorded in our samples (1.31-1-54) were lower than those reported for pistachios from mature orchards in other regions, including values of 3.8–4.5 described by Ouni et al. [43], 2.40–3.66 described by Esteki et al. [46] and 1.9–3.7 described by Tsantili et al. [11], which could affect the fat stability of pistachios from young trees. These differences likely reflect the combined influence of tree age, cultivar, and environmental conditions.

The saturated fatty acids (SFAs) ranged from 16.48–24.80%; monounsaturated fatty acids (MUFAs) ranged from 42.91–50.59%; polyunsaturated fatty acids (PUFAs) ranged from 32.34–33.18%. The unsaturated/saturated fatty acids ratios varied between 3.37 and 4.52. The results obtained differ from earlier reports in the literature [46]. We found high palmitic acid and a lower oleic acid concentration; these affect both the unsaturated/saturated and the O/L ratio.

Climatic factors have been related to the differences found in the concentrations of fatty acids in pistachios. Low temperatures have been suggested as responsible for higher palmitic acid and lower oleic acid contents [28]. This would explain our results as the since temperatures in the Castilla y León region are lower than in other pistachio producing countries.

The results in Table 5 show that only the linolenic acid was significantly (*p* = 0.001) affected by the treatments, with higher values observed in the treatments incorporating compost tea (T2 and T3). In 2022 treatments 2 and 3 produced significantly higher values of this fatty acid than the control treatment. This suggests that incorporating compost tea could affect the linolenic acid content. However, in 2023 only T2 produced a higher, but not significantly different, linoleic acid content. In other crops compost and compost tea have been reported to produce a significantly positive impact on nutritional status and gamma linolenic acid content [47]. In *Borago officinalis*, treatment with compost tea increased the linolenic acid content of the vegetable oil [48]. In addition, Li et al. [49] demonstrated linolenic acid content is enhanced in tomatoes treated with compost tea.

The growing season also affected the concentrations of oleic acid and the oleic/linoleic O/L ratio. These parameters were lower in 2022 while all other fatty acids had higher concentrations. This resulted in a much higher SFA content compared with year 2023. For all fatty acids analyzed, the growing season had a significant effect on their composition (*p* < 0.05). Climatic conditions must also be taken into consideration when analyzing the fatty acid profile. Amaral et al. [50] in hazelnut and Karaat [51] in almond reported that fatty acid composition is significantly influenced by environmental variables, with the most notable differences occurring in oleic and linoleic acid contents. Satil et al. [28] reported that warmer climates produce pistachios with a higher saturated fat concentration. In our study, a decrease in both spring and summer rainfall was observed in the 2023 season with higher temperatures in spring and summer (Table A1), which could account for the higher oleic and linoleic concentrations observed in 2023.

### 3.5. Amino Acids Profile

The amino acid composition is detailed in Table 6, (g 100 g^−1^) of pistachios as a function of treatments and years. Glutamic acid (4.82–5.75 g 100 g^−1^) was the most abundant amino acid in all pistachio nuts analyzed, followed by arginine (2.13–2.93 g 100 g^−1^) and aspartic acid (2.14–2.46 g 100 g^−1^), irrespective of fertilization treatment and season. The amino acids found in lower concentrations were methionine (0.25–0.70 g 100 g^−1^), histidine (0.55–0.97 g 100 g^−1^) and threonine (0.77–1.09 g 100 g^−1^). In the 2023 growing season histidine concentration was below the quantification limit of the method (LOQ 0.05 g 100 g^−1^) and therefore could not be reliably quantified.

Mahmoodabadi et al. [52] found that glutamic acid, aspartic acid, and arginine were identified as the major amino acids in pistachio. However, Derbyshire et al. [53] describes arginine as the major amino acid in pistachio followed by leucine. The amounts of amino acids found in our pistachios were similar to the levels reported by Chung et al. [54], Bailey and Stein [55] and Derbyshire et al. [53]. The total protein content of pistachios (Table 3) was between 22.28 and 25.18 g 100 g^−1^, indicating that they constitute a good source of protein. Although the protein content of pistachio nuts is adequate, its biological value is less clear according to Brufau et al. [56]. The presence of essential amino acids is limited, with low concentrations of leucine and histidine. However, Derbyshire et al. [53] qualify the pistachio as a complete protein as it provides the 9 essential amino acids (EAAs). Pistachio proteins are rich in arginine and low in lysine, the opposite of animal proteins, making them healthier Kudlackova et al. (2005) [57]. In our pistachios, the lysine/arginine ratio was in the range 0.53 and 0.6, consistent with Souci et al.’s findings [58].

When the amino acid content was reviewed there were no differences among mineral fertilization, organic or mixed fertilization. For all amino acids, lower contents were observed in the T2 and T3 treatments, except for histidine and glutamic acid; however, these differences were not statistically significant (Appendix A—Table A3). This lack of significance is probably due to the large variability found among batches analyzed within the same treatment, as can be seen in the high deviations (Table 6). These results seem to indicate that, even within the same orchard, amino acid content changes significantly from tree to tree. Histidine content in kernels from the 2023 growing season was below the detection limit. Although this does not affect the overall interpretation of the amino acid profile, it should be considered when assessing the completeness of individual amino acid data for that season.

Lower contents of serine, histidine, glycine, valine, leucine and isoleucine were observed in 2023 growing season, which is consistent with the lower total protein content of these pistachios (Table 3). The GS factor was significant for serine, histidine, glycine and valine. The difficult climatic conditions in 2023 were associated with reduced contents of protein, sugars, ash and minerals and the amino acids.

The influence that climate or the year of cultivation have on the amino acid composition of pistachio has not been studied. However, based on the results obtained in this study, this aspect warrants further investigation, particularly in light of the importance of the amino acid composition in consumers’ diets. As consumers are demanding new sources of plant-based protein, pistachio described as a balanced and good quality source of plant protein [53], could be valuable dietary option.

### 3.6. Multivariate Statistical Analysis

**Figure 1:**
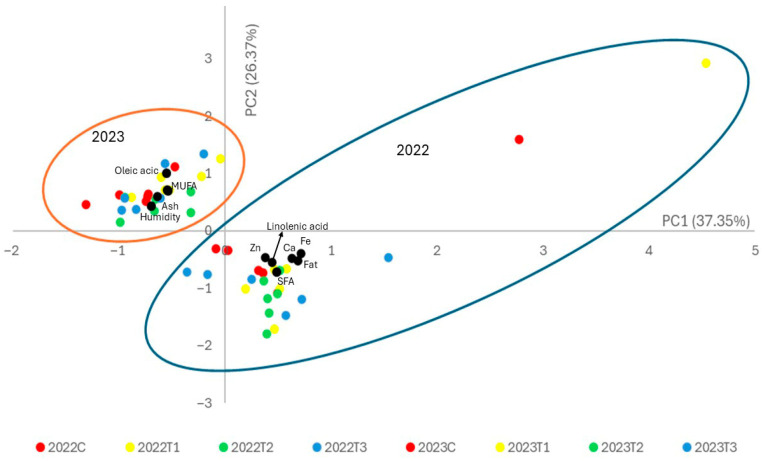
Score plot of pistachio nut samples in the space defined by the first two principal components.

Principal component analysis (PCA) was conducted to assess variation and relationships among the studied variables. Variables that did not obtain a value greater than 0 for all analyzed samples were eliminated from the analysis. Figure 1 shows the diagram of the samples and the variables with the greatest weight. PC1, which is the most important component, represents 37.35% of the total variation, while PC2 represented 26.37% of the total variation. Although the model showed limited predictive accuracy with a low threshold for a robust fit [59], it allowed the identification of non-directed data sets.

Figure 1 shows a clear grouping of the samples analyzed by year of cultivation. Overall, the results highlight the relevance of interannual climatic variability in modulating pistachio kernel composition during early bearing. Differences between growing seasons largely precipitated the observed variability in nut composition. However, the effects of fertilization treatments were more subtle and dependent on the environmental conditions each season. Greater variability among the pistachio samples was observed in 2022, when, as mentioned above, yields were lower and the resulting composition of the fruits had a higher ash and sugar content and a lower fat content (Table 2). Considering the treatments applied in 2023 had less variability, it is not possible to establish differences between treatments. However, in 2022 the compost tea treatments (T2 and T3) produced differences relative to the control. Among the variables affected were linolenic (−0.540), palmitic (−0.721) and palmitoleic (−0.607) fatty acids, which contributed to PC2.

## 4. Conclusions

This study evaluated the response of pistachio trees during early bearing to different fertilization strategies: mineral fertilization (T0), compost (T1), compost tea (T2), and their combined application with mineral fertilization (T3). Both agronomic and nut composition parameters were investigated. Treatments T1, T2, and T3 had comparable vegetative growth, yield, and nut quality relative to the mineral control. This suggests compost-based amendments can be effectively integrated into pistachio fertilization programs without compromising productivity or kernel composition. Although the combined treatment (T3) produced stable performance across the two seasons, the results also demonstrated that interannual climatic variability exerted a stronger influence on yield and kernel composition than the treatments. Therefore, while these findings provide valuable insights into the responses of pistachio trees in the early bearing years, longer-term field trials are required to confirm the feasibility of these results for commercial pistachio production.

## Figures and Tables

**Table 1:** Physicochemical characteristics of the compost tea (assimilable nutrients).

pH	Ec (dSm^−1^)	NO_3_ (mg kg^−1^)	P_2_O_5_ (mg kg^−1^)	K_2_O (mg kg^−1^)	SO_4_^2−^ (mg kg^−1^)
7.32 ± 0.14	1.22 ± 0.11	3200 ± 185	102 ± 65	3840 ± 320	28 ± 16
**Humic Acids (mg kg^−1^)**	**Ca (mg kg^−1^)**	**Mg (mg kg^−1^)**	**Fe (mg kg^−1^)**	**B (mg kg^−1^)**	**Zn (µg kg^−1^)**
190 ± 40	146 ± 32	150 ± 39	9.8 ± 2.1	17.2 ± 6.3	2.66 ± 2.5

**Table 2:** Growth-Production parameters and organic fertilization incidence on *Pistacia vera* v. Kerman trees under three different treatments for the two different growing seasons (mean ± SD).

GS	T	Chlorophyll Content (SPAD Unit)	Shoot Length (cm)	Trunk Diameter Growth * (cm)	Average Weight Shell Nuts (g)	Average Weight Kernels (g)	Shell Nut Yield (kg Tree^−1^)	Kernels Nut Yield (kg Tree^−1^)
**2022**	**C**	41.27 ± 0.54 a	21.00 ± 6.89 a	0.97 ± 0.36 a	2.33 ± 0.59 a	1.71 ± 0.42 b	1.86 ± 0.51 a	1.22 ± 0.34 a
	**T1**	41.84 ± 0.62 a	38.00 ± 8.58 b	0.93 ± 0.39 a	2.33 ± 0.65 a	1.71 ± 0.50 b	1.70 ± 0.50 a	1.09 ± 0.30 a
	**T2**	42.01 ± 0.61 a	25.90 ± 9.38 ab	1.01 ± 0.40 a	2.21 ± 0.75 a	1.53 ± 0.55 a	1.53 ± 0.72 a	0.97 ± 0.48 a
	**T3**	42.29 ± 0.78 a	23.80 ± 5.54 a	1.04 ± 0.62 a	2.31 ± 0.68 a	1.65 ± 0.50 ab	1.43 ± 0.71 a	0.93 ± 0.48 a
**2023**	**C**	43.21 ± 0.54 ab	37.20 ± 3.7 a	0.81 ± 0.61 a	1.45 ± 0.34 ab	1.11 ± 0.26 ab	2.27 ± 0.32 b	1.48 ± 0.32 b
	**T1**	43.92 ± 0.58 b	34.10 ± 4.24 a	0.61 ± 0.27 a	1.33 ± 0.41 a	1.03 ± 0.30 a	1.93 ± 0.17 ab	1.27 ± 0.23 ab
	**T2**	41.43 ± 1.10 a	34.20 ± 3.23 a	0.76 ± 0.41 a	1.39 ± 0.36 a	1.08 ± 0.27 a	1.70 ± 0.48 a	1.11 ± 0.34 a
	**T3**	43.13 ± 0.45 ab	37.80 ± 1.84 a	0.67 ± 0.40 a	1.57 ± 0.41 b	1.20 ± 0.30 b	2.13 ± 0.28 b	1.41 ± 0.33 b
**Significance**								
**T**		0.493	0.034	0.533	0.052	0.004	0.024	0.018
**GS**		0.028	<0.001	<0.001	<0.001	<0.001	<0.001	0.001
**T × GS**		0.130	0.001	0.6329	0.124	0.071	0.252	0.279

* Measured between February and October. T: treatment, GS: growing season. Fertilization treatments included traditional mineral fertilization (C), compost (T1), compost combined with compost tea (T2), and compost tea combined with traditional fertilization (T3). a, b Different letters for each parameter within the same growing season (GS), mean values showing statistically significant differences (*p* < 0.05) were identified using Tukey’s HSD test.

**Table 3:** Proximate composition (g 100 g^−1^ dw) of pistachios in each harvest season and the average value of the two growing seasons.

GS	T	Fat	Protein	Total Carbohydrates	Fibre	Starch	Total Sugars	Ash	Energy (kcal/100 g)
**2022**	**C**	48.53 ± 0.63 ab	24.02 ± 1.37 a	24.13 ± 1.42 a	10.42 ± 1.32 a	3.71 ± 0.32 a	9.23 ± 0.16 b	3.32 ± 0.06 a	647.22 ± 2.07 b
	**T1**	49.87 ± 0.62 c	25.18 ± 1.28 a	21.58 ± 1.51 a	10.52 ± 1.37 a	3.52 ± 0.34 a	9.15 ± 0.25 ab	3.38 ± 0.04 a	649.84 ± 2.31 b
	**T2**	49.56 ± 0.64 bc	24.20 ± 2.68 a	22.83 ± 2.87 a	11.33 ± 3.14 a	3.71 ± 0.15 a	8.80 ± 0.13 a	3.41 ± 0.08 a	645.63 ± 1.54 ab
	**T3**	48.06 ± 0.71 a	24.57 ± 0.65 a	23.96 ± 1.00 a	10.64 ± 1.40 a	3.68 ± 0.22 a	9.07 ± 0.41 ab	3.42 ± 0.05 a	642.24 ± 4.93 a
**2023**	**C**	51.67 ± 0.39 ab	22.90 ± 0.96 a	22.66 ± 0.75 a	10.21 ± 0.50 a	4.11 ± 0.50 a	8.14 ± 0.24 a	2.77 ± 0.05 a	629.35 ± 3.36 a
	**T1**	52.26 ± 0.45 b	22.28 ± 0.72 a	22.61 ± 1.06 a	10.52 ± 0.80 a	4.33 ± 0.41 a	7.91 ± 0.14 a	2.86 ± 0.04 a	635.84 ± 3.02 c
	**T2**	51.39 ± 0.31 ab	22.91 ± 1.09 a	22.86 ± 1.24 a	10.51 ± 1.32 a	4.46 ± 0.14 a	8.16 ± 0.28 a	2.84 ± 0.04 a	634.14 ± 3.21 bc
	**T3**	50.83 ± 0.98 a	22.60 ± 2.19 a	23.59 ± 2.53 a	10.47 ± 0.71 a	4.28 ± 0.27 a	7.93 ± 0.21 a	2.97 ± 0.08 b	626.64 ± 3.40 ab
Significance									
**T**		<0.001	0.979	0.102	0.913	0.503	0.174	<0.001	<0.001
**GS**		<0.001	<0.001	0.692	0.606	<0.001	<0.001	<0.001	<0.001
**T** × **GS**		0.084	0.479	0.358	0.980	0.411	0.022	0.054	0.103

GS: growing season, T: Treatment; C: Traditional fertilization, T1: Fertilization with compost, T2: Fertilization with compost combined with compost tea, T3: Fertilization with compost tea combined with traditional fertilization. Different letters, a, b, c, within the same GS, mean values showing statistically significant differences (*p* < 0.05) were identified using Tukey’s HSD test.

**Table 4:** Mineral composition analysis (mg kg^−1^) of pistachios over two harvest seasons.

GS	T	Mg	Ca	Fe	Zn
**2022**	**C**	1124.54 ± 74.22 a	1207.24 ± 88.24 a	54.37 ± 8.85 a	16.68 ± 2.57 a
	**T1**	1198.56 ± 52.24 a	1208.54 ± 90.94 a	55.60 ± 7.18 a	17.74 ± 2.88 a
	**T2**	1145.01 ± 63.02 a	1176.59 ± 51.42 a	51.56 ± 3.84 a	19.36 ± 4.00 ab
	**T3**	1183.35 ± 71.42 a	1216.80 ± 66.01 a	54.44 ± 5.87 a	23.56 ± 2.81 b
**2023**	**C**	1118.01 ± 64.53 a	1032.00 ± 45.54 a	40.64 ± 5.18 a	12.65 ± 1.27 a
	**T1**	1114.21 ± 103.92 a	1043.34 ± 44.13 ab	38.15 ± 4.04 a	15.15 ± 3.99 a
	**T2**	1194.80 ± 89.44 a	1088.84 ± 5.77 b	42.61 ± 5.51 a	16.45 ± 3.91 a
	**T3**	1136.81 ± 70.73 a	1083.11 ± 41.27 b	49.32 ± 7.79 a	16.39 ± 2.80 a
Significance					
**T**		0.310	0.629	0.460	0.006
**GS**		0.400	<0.001	<0.001	<0.001
**T** × **GS**		0.080	0.297	0.410	0.110

GS: growing season, T: Treatment, C: Traditional fertilization, T1: Fertilization with compost, T2: Fertilization with compost combined with compost tea, T3: Fertilization with compost tea combined with traditional mineral fertilization. Different letters, a, b within the same GS, mean values showing statistically significant differences (*p* < 0.05) were identified using Tukey’s HSD test.

**Table 5:** Fatty acid profile of pistachios during two harvest seasons. The results were expressed as percentage of total fatty acid methyl esters.

	T	Palmitic Acid (C16:0)	Palmitoleic Acid (C16:1)	Estearic Acid (C18:0)	Oleic Acid (C18:1)	Linoleic Acid (C18:2)	Linolenic Acid (C18:3)	Oleic/Linoleic Ratio	SFAs	MUFAs	PUFAs
**2022**	**C**	19.82 ± 1.56 a	1.36 ± 0.20 a	2.96 ± 0.84 a	43.42 ± 1.75 a	31.85 ± 1.60 a	0.60 ± 0.28 a	1.36 ± 0.07 a	22.78 ± 1.52 a	44.78 ± 1.62 a	32.45 ± 0.69 a
	**T1**	18.64 ± 1.09 a	1.64 ± 0.09 a	6.16 ± 8.21 a	44.36 ± 1.94 a	31.35 ± 0.86 a	0.99 ± 0.15 ab	1.41 ± 0.10 a	24.80 ± 7.64 a	46.00 ± 2.02 a	32.34 ± 0.98 a
	**T2**	20.81 ± 0.84 a	1.93 ± 0.67 a	3.75 ± 0.75 a	40.97 ± 2.42 a	31.37 ± 1.31 a	1.16 ± 0.09 b	1.31 ± 0.13 a	24.57 ± 1.51 a	42.91 ± 2.38 a	32.52 ± 1.30 a
	**T3**	20.01 ± 2.02 a	1.54 ± 0.10 a	3.52 ± 0.98 a	42.18 ± 4.77 a	31.59 ± 1.60 a	1.17 ± 0.40 b	1.34 ± 0.22 a	23.52 ± 3.00 a	43.72 ± 4.85 a	32.76 ± 1.92 a
**2023**	**C**	15.17 ± 0.48 a	1.01 ± 0.09 a	1.49 ± 0.06 a	49.16 ± 0.44 a	32.52 ± 0.87 a	0.66 ± 0.06 a	1.51 ± 0.05 a	16.66 ± 0.52 a	50.17 ± 0.44 a	33.18 ± 0.92 a
	**T1**	15.45 ± 0.29 a	1.16 ± 0.06 a	1.52 ± 0.05 a	49.16 ± 0.61 a	32.01 ± 0.60 a	0.70 ± 0.02 a	1.54 ± 0.05 a	16.97 ± 0.30 a	50.32 ± 0.57 a	32.71 ± 0.62 a
	**T2**	15.06 ± 0.51 a	1.17 ± 0.11 a	1.42 ± 0.07 a	49.42 ± 0.46 a	32.22 ± 0.51 a	0.71 ± 0.02 a	1.53 ± 0.03 a	16.48 ± 0.57 a	50.59 ± 0.37 a	32.93 ± 0.50 a
	**T3**	15.38 ± 0.56 a	1.09 ± 0.09 a	1.25 ± 0.13 a	49.16 ± 0.88 a	32.49 ± 0.90 a	0.64 ± 0.05 a	1.51 ± 0.07 a	16.62 ± 0.49 a	50.25 ± 0.83 a	33.13 ± 0.93 a
**Significance**											
**T**		0.237	0.011	0.528	0.313	0.580	0.001	0.581	0.793	0.402	0.804
**GS**		<0.001	<0.001	0.003	<0.001	0.010	<0.001	<0.001	<0.001	<0.001	0.137
**T × GS**		0.048	0.249	0.591	0.199	0.986	0.002	0.659	0.850	0.272	0.970

T: treatment; GS: growing season, C: Traditional fertilization, T1: Fertilization with compost, T2: Fertilization with compost combined with compost tea, T3: Fertilization with compost tea combined with traditional fertilization, SFA: saturated fatty acids, MUFA: monounsaturated fatty acids, PUFA: polyunsaturated fatty acids. Different letters, a, b within the same GS, mean values showing statistically significant differences (*p* < 0.05) were identified using Tukey’s HSD test.

**Table 6:** Amino acid composition (g 100 g^−1^) of pistachios during two harvest seasons.

		Control	T1	T2	T3
Aspartic acid	2022	2.28 ± 0.15 a	2.35 ± 0.37 a	2.15 ± 0.14 a	2.28 ± 0.38 a
	2023	2.14 ± 0.18 a	2.46 ± 0.31 a	2.25 ± 0.26 a	2.30 ± 0.31 a
Glutamic acid	2022	4.82 ± 1.00 a	5.34 ± 0.49 a	5.18 ± 0.39 a	5.49 ± 0.99 a
	2023	4.98 ± 0.48 a	5.75 ± 7.01 a	5.26 ± 0.56 a	5.41 ± 0.73 a
Serine	2022	1.53 ± 0.23 a	1.84 ± 0.92 a	1.42 ± 0.11 a	1.47 ± 0.26 a
	2023	1.28 ± 0.12 a	1.43 ± 0.13 a	1.31 ± 0.09 a	1.33 ± 0.14 a
Histidine	2022	0.96 ± 0.97 a	0.69 ± 0.40 a	0.97 ± 0.85 a	0.55 ± 0.08 a
	2023	nd	nd	nd	nd
Glycine	2022	1.12 ± 0.15 a	1.28 ± 0.58 a	1.02 ± 0.06 a	1.06 ± 0.70 a
	2023	0.93 ± 0.08 a	0.98 ± 0.08 a	0.90 ± 0.05 a	0.94 ± 0.10 a
Threonine	2022	1.02 ± 0.61 a	0.99 ± 0.52 a	0.77 ± 0.05 a	0.80 ± 0.13 a
	2023	0.98 ± 0.16 a	1.09 ± 0.13 a	0.95 ± 0.06 a	0.99 ± 0.11 a
Arginine	2022	2.51 ± 0.41 a	2.93 ± 1.55 a	2.19 ± 0.17 a	2.29 ± 0.41 a
	2023	2.15 ± 0.18 a	2.42 ± 0.21 a	2.13 ± 0.15 a	2.26 ± 0.38 a
Alanine	2022	1.16 ± 0.28 a	1.23 ± 0.59 a	0.97 ± 0.08 a	1.00 ± 0.18 a
	2023	1.00 ± 0.09 a	1.09 ± 0.09 a	0.99 ± 0.07 a	1.05 ± 0.12 a
Tyrosine	2022	1.10 ± 1.14 a	0.85 ± 0.48 a	0.64 ± 0.05 a	0.67 ± 0.12 a
	2023	0.67 ± 0.05 a	0.73 ± 0.05 a	0.63 ± 0.06 a	0.71 ± 0.15 a
Valine	2022	1.45 ± 0.38 a	1.72 ± 1.03 a	1.28 ± 0.11 a	1.33 ± 0.25 a
	2023	1.05 ± 0.13 a	1.13 ± 0.10 a	0.98 ± 0.07 a	1.06 ± 0.18 a
Methionine	2022	0.70 ± 1.04 a	0.37 ± 0.21 a	0.25 ± 0.05 a	0.29 ± 0.05 a
	2023	0.52 ± 0.10 a	0.49 ± 0.06 a	0.42 ± 0.08 a	0.49 ± 0.11 a
Phenylalanine	2022	1.47 ± 0.81 a	1.48 ± 0.82 a	1.12 ± 0.09 a	1.17 ± 0.21 a
	2023	1.25 ± 0.11 a	1.35 ± 0.14 a	1.21 ± 0.10 a	1.29 ± 0.18 a
Isoleucine	2022	1.21 ± 0.62 a	1.25 ± 0.70 a	0.94 ± 0.08 a	0.98 ± 0.17 a
	2023	0.93 ± 0.09 a	1.08 ± 0.12 a	0.95 ± 0.09 a	0.99 ± 0.14 a
Leucine	2022	1.80 ± 0.34 a	2.09 ± 1.05 a	1.64 ± 0.12 a	1.70 ± 0.29 a
	2023	1.61 ± 0.16 a	1.78 ± 0.16 a	1.60 ± 0.12 a	1.67 ± 0.21 a
Lysine	2022	1.60 ± 0.58 a	1.76 ± 1.03 a	1.35 ± 0.09 a	1.41 ± 0.17 a
	2023	1.26 ± 0.12 a	1.50 ± 0.13 b	1.38 ± 0.10 ab	1.35 ± 0.12 ab

T1: Fertilization with compost, T2: Fertilization with compost combined with compost tea, T3: Fertilization with compost tea combined with traditional fertilization. nd: concentrations below the detection limit of the method. Different letters, a, b within the same GS, mean values showing statistically significant differences (*p* < 0.05) were identified using Tukey’s HSD test.

## Data Availability

Data supporting reported results can be found in the GREDOS repository of Universidad de Salamanca. http://hdl.handle.net/10366/168114 (accessed on 10 January 2026).

## Appendix A

**Table A1:** Meteorological data for the growing area for the years 2022 and 2023. Obtained from the online platform INFORIEGO (www.inforiego.org) for the locality of Parada de Rubiales (Salamanca, Spain).

Season	Temperature (°C)			Humidity (%)			Wind Speed (m/s)	Radiation (MJ/m^2^)	Precipitation (mm)
	$\overset{-}{x}$	max	min	$\overset{-}{x}$	max	min			
2022									
Winter	5.14	11.02	0.35	77.34	92.48	54.46	0.69	8.87	98.23
Spring	10.71	16.78	4.68	65.78	89.33	39.90	0.89	19.61	155.45
Summer	21.31	30.18	12.26	48.63	80.94	21.64	0.66	27.52	23.81
Autumn	13.27	19.84	7.70	69.57	89.41	43.41	0.60	12.90	123.20
Annual	12.61	19.46	6.25	65.33	88.04	39.85	0.71	17.22	400.69
2023									
Winter	3.05	7.39	−0.54	87.23	96.05	70.67	0.59	6.60	60.44
Spring	11.61	18.64	4.70	58.50	84.41	32.08	0.77	21.88	56.91
Summer	20.34	28.58	12.20	55.13	84.32	26.51	0.45	27.46	32.69
Autumn	13.36	19.72	8.10	72.50	90.18	47.22	0.55	12.58	321.06
Annual	12.09	18.58	6.12	68.34	88.74	44.12	0.59	17.13	471.10

**Table A2:** Calibration curves for quantifying mineral content.

Compound	Calibration Curve	R	DL	BEC
Ca	y = 0.0166 * x + 7.7514 × 10^−5^	1.000	0.01333	0.00466
Mg	y = 5.5709 * x + 0.0015	1.000	5.722 × 10^−5^	0.0002731
Fe	y = 0.0661 * x + 0.0641	1.000	0.04303	0.9701
Zn	y = 0.0205 * x + 0.0034	1.000	0.03054	0.1679

R: Pearson correlation coefficient. DL: Limit of detection (mg kg^−1^ for Ca and Mg and µg kg^−1^ for Fe and Zn). BEC: Background Equivalent Concentration. * Units differ depending on the element, as specified above.

**Table A3:** Statistical results for the amino acid composition of pistachio nuts.

		Coefficient of Variation (%)				Significance		
		Control	T1	T2	T3	T	GS	T × GS
Aspartic acid	2022	6.40	15.78	6.67	16.88	0.260	0.799	0.670
	2023	8.38	12.58	11.40	13.26			
Glutamic acid	2022	20.82	59.96	7.58	18.16	0.160	0.578	0.685
	2023	9.60	12.20	10.71	13.41			
Serine	2022	15.36	50.27	7.53	17.38	0.257	0.040	0.746
	2023	9.17	9.07	6.98	10.43			
Histidine	2022	100.64	58.13	87.07	15.19	0.633	<0.001	0.633
	2023	-	-	-	-			
Glycine	2022	13.01	45.35	5.98	15.95	0.309	0.008	0.738
	2023	8.70	8.03	5.16	10.11			
Threonine	2022	59.71	52.40	6.37	15.89	0.394	0.220	0.786
	2023	16.57	12.24	5.89	10.60			
Arginine	2022	16.21	53.11	7.92	17.86	0.220	0.187	0.727
	2023	8.59	8.68	7.21	16.69			
Alanine	2022	24.00	48.16	7.94	18.09	0.364	0.428	0.655
	2023	9.12	7.95	6.80	11.31			
Tyrosine	2022	104.10	56.94	7.71	17.98	0.532	0.336	0.569
	2023	7.09	7.33	9.95	21.15			
Valine	2022	26.02	59.65	8.46	18.56	0.333	0.002	0.768
	2023	12.61	8.76	7.54	16.97			
Methionine	2022	149.89	56.48	20.30	16.48	0.339	0.460	0.598
	2023	18.95	11.95	19.77	22.01			
Phenylalanine	2022	55.39	55.43	8.09	17.70	0.449	0.772	0.731
	2023	9.14	10.50	8.35	13.86			
Isoleucine	2022	51.09	56.08	8.16	17.47	0.435	0.284	0.667
	2023	9.95	10.83	9.27	14.15			
Leucine	2022	18.67	50.15	7.61	16.96	0.288	0.246	0.818
	2023	9.83	9.04	7.67	12.53			
Lysine	2022	36.08	58.65	6.68	12.30	0.423	0.217	0.712
	2023	9.69	8.97	7.38	9.02			

T: treatment; GS: growing season, Control: Traditional fertilization, T1: Fertilization with compost, T2: Fertilization with compost combined with compost tea, T3: Fertilization with compost tea combined with traditional fertilization.

**Table A4:** Limit of quantification (LoQ) of the analytical methods and equipment used for the quantification of proximate composition and amino acid content and Background Equivalent Concentration (BEC) of the mineral analysis.

Compound	LoQ (g 100 g^−1^)
Fat	0.5
Protein	0.4
Fibre	1
Starch	0.5
Total sugars	1
Ash	0.25
Amino acids	0.05
	BEC
Mg	0.0002731 mg kg^−1^
Ca	0.00466 mg kg^−1^
Zn	0.1679 µg kg^−1^
Fe	0.9701 µg kg^−1^
